# Enabling uncertainty estimation in neural networks through weight perturbation for improved Alzheimer's disease classification

**DOI:** 10.3389/fninf.2024.1346723

**Published:** 2024-02-06

**Authors:** Matteo Ferrante, Tommaso Boccato, Nicola Toschi

**Affiliations:** ^1^Department of Biomedicine and Prevention, University of Rome Tor Vergata, Rome, Italy; ^2^Martinos Center for Biomedical Imaging, Massachusetts General Hospital and Harvard Medical School, Boston, MA, United States

**Keywords:** Bayesian, uncertainty, deep learning, convolutional neural network (CNN), classification

## Abstract

**Background:**

The willingness to trust predictions formulated by automatic algorithms is key in a wide range of domains. However, a vast number of deep architectures are only able to formulate predictions without associated uncertainty.

**Purpose:**

In this study, we propose a method to convert a standard neural network into a Bayesian neural network and estimate the variability of predictions by sampling different networks similar to the original one at each forward pass.

**Methods:**

We combine our method with a tunable rejection-based approach that employs only the fraction of the data, i.e., the share that the model can classify with an uncertainty below a user-set threshold. We test our model in a large cohort of brain images from patients with Alzheimer's disease and healthy controls, discriminating the former and latter classes based on morphometric images exclusively.

**Results:**

We demonstrate how combining estimated uncertainty with a rejection-based approach increases classification accuracy from 0.86 to 0.95 while retaining 75% of the test set. In addition, the model can select the cases to be recommended for, e.g., expert human evaluation due to excessive uncertainty. Importantly, our framework circumvents additional workload during the training phase by using our network “turned into Bayesian” to implicitly investigate the loss landscape in the neighborhood of each test sample in order to determine the reliability of the predictions.

**Conclusion:**

We believe that being able to estimate the uncertainty of a prediction, along with tools that can modulate the behavior of the network to a degree of confidence that the user is informed about (and comfortable with), can represent a crucial step in the direction of user compliance and easier integration of deep learning tools into everyday tasks currently performed by human operators.

## Highlights

- Uncertainty estimation key point to implement deep learning in clinical settings.
- A neural network can be used to probe the local confidence in inference.
- No extra computational time or challenging settings.
- Saying “I don't know” and asking for human evaluation can improve trust and usability of automatic systems.
- Up to +10% improvement in performance for AD classification from morphometrics images over 75% of dataset.

## 1 Introduction

The willingness to trust predictions formulated by automatic algorithms is key in a wide range of domains. In addition to questions of ethics and responsibility, it is important to note that, while extremely powerful, a vast number of deep architectures are only able to formulate predictions without an associated uncertainty. This shortcoming critically reduces user compliance even when explainability techniques are used, and this issue is particularly sensitive when deep learning techniques are employed, e.g., in the medical diagnosis field. Producing a measure of the system's confidence in its prediction can significantly improve trustability in the deep learning tool as a recommendation machine, which is capable of improving the workflow of physicians. Alzheimer's disease (AD) is one of the most critical public health concerns of our time. Due to the increase in life expectancy and better professional care, more and more people reach older ages but are often affected by degenerative brain disorders such as AD, which is a severe form of dementia (Knopman et al., 2021). The main symptoms are progressive memory loss, difficulties in normal life activities, language disorders, disorientation, and, in general, decreased cognitive functions. Although in some cases specific genetic mutations are responsible for the onset of the disease, one of the most important risk factors is age (which, however, can also be related to comorbidities).

As a progressive degenerative pathology, AD is usually preceded by a different condition called mild cognitive impairment (MCI), with less intense symptoms that often, but not always, evolve into AD, which has no cure.

There are many theories about the etiopathogenesis of AD, several of which are linked to an alteration in the metabolism of beta-amyloid precursor protein. The causal relationship between beta-myeloid metabolism and the clinical presentation of AD is the subject of intense research (Hampel et al., 2018, 2020; Spasov et al., 2019; Toschi et al., 2019). In clinical practice, AD diagnosis is based on symptoms and is commonly confirmed using magnetic resonance imaging (MRI) or, in some cases, positron emission tomography (PET), leaving the clinician with a great deal of subjectivity and uncertainty to deal with when positioning a patient in the AD continuum. For these reasons, there is great interest in models capable of detecting and predicting AD-related structural and functional changes. Deep learning models are able to usefully extract local and global characteristics through convolutional layers and learn how to predict interesting outcomes, such as distinguishing healthy controls from AD patients or even MCI patients, which will remain stable for those who will progress to AD (Jo et al., 2019; Spasov et al., 2019; Sethi et al., 2022; Termine et al., 2022). In this context, difficulties in accessing large-scale curated datasets and the need to work with multimodal high-dimensional data call for particular attention in avoiding overfitting and increasing the reliability of automatic models, possibly including the output of uncertainty estimates, which can be evaluated by neuroscientists and physicians.

**Figure 1:**
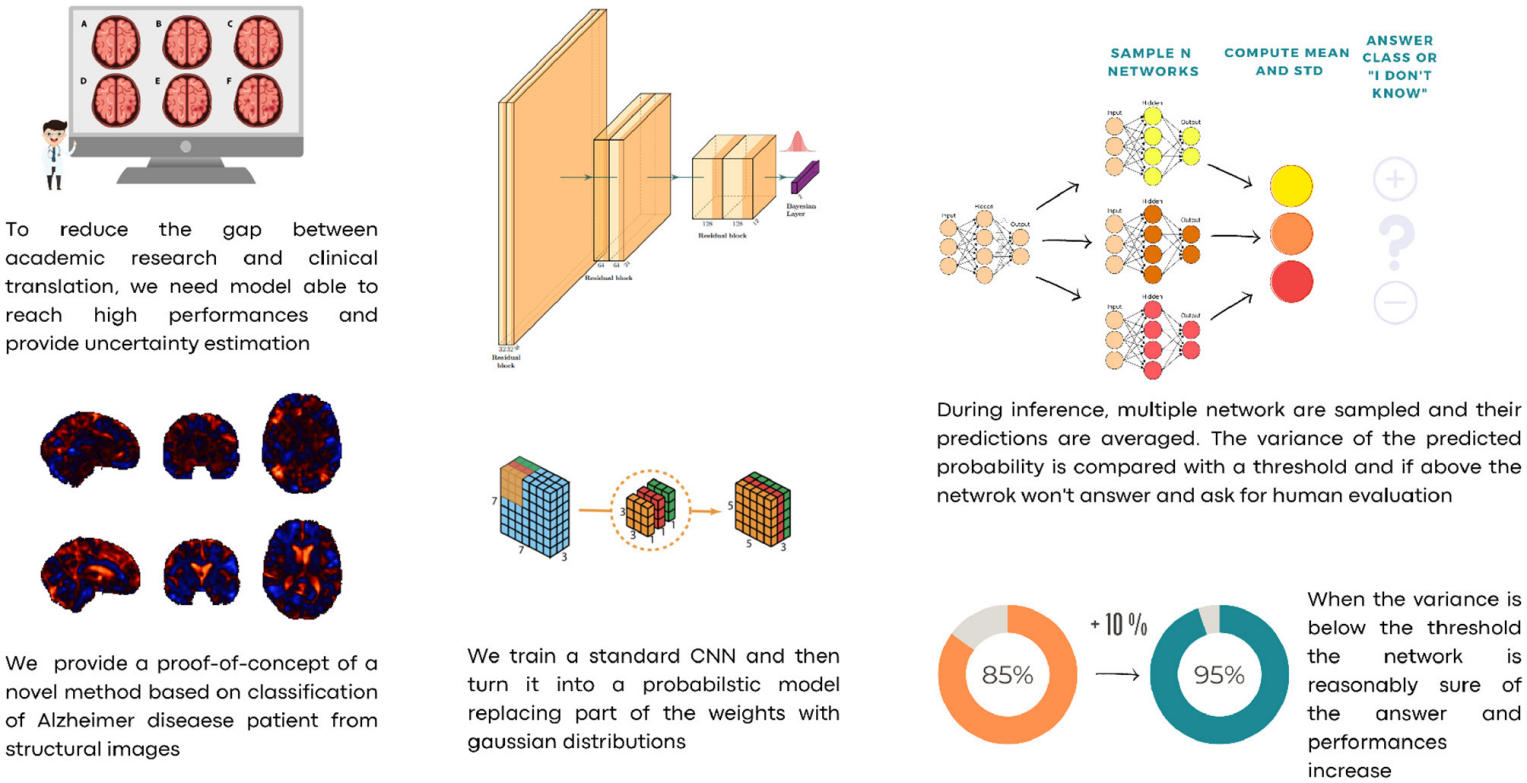
A schematic description of our approach.

For those reasons, we propose a Hybrid Bayesian Neural Network in a framework where predicted probabilities are coupled with their uncertainties. To reduce the number of parameters, we propose a convolutional neural network based on depth-wise separable convolutions. We train our model on a subset of the Alzheimer's Disease Neuroimaging Initiative (ADNI) dataset using Jacobian Determinant images, that is, images where each voxel describes the change in volume element resulting from non-linear coregistration of the patient's structural magnetic resonance imaging of the brain to a standard space, e.g., the Montreal Neurological Institute (MNI) T1-weighted template. This choice was made to isolate morphometric changes (e.g., cortical atrophy) from the image intensity variations (Hua et al., 2008). Once the model is trained, we turn the last linear layer into a Hybrid Bayesian neural network, replacing optimal values w* with a narrow parameter distribution $N\left(w^{*},s\right)$. This means that instead of having a single weight value for each connection between two neurons from the last layer to the final output, a Gaussian distribution centered on the optimal value w^*^ is used. Every time the network makes an inference, the weights of the last layer are sampled from those distributions. In this way, it is possible to obtain *N* slightly different networks which, in turn, allow the network to perform ensembling and hence provide an uncertainty estimate. The latter can also be thresholded to subset the data and increase prediction performance. Our approach allows us to employ a straightforward cost function for the classification problem while estimating sample-wise uncertainty at inference time. Unlike other methods such as Monte Carlo Dropout (Gal and Ghahramani, 2016) or standard Bayesian Neural Networks, we do not introduce uncertainty by altering the network's architecture or weight distribution, both of which can lead to slower convergence, more difficult training, and lower performance. Instead, for each sample, we investigate the loss landscape vicinity at inference time to evaluate the quality and uncertainty of predictions. In the case of excessive uncertainty, this can trigger a request for human verification. Figure 1 shows a scheme of the overall procedure.

In the field of Alzheimer's disease classification, various methodologies have been explored, including the integrative analysis of the hippocampus and amygdala using shape and diffusion tensor imaging, as presented in the studies mentioned in Tang et al. (2016) and Spasov et al. (2019). These approaches highlight the potential of morphometric and microstructural features in distinguishing Alzheimer's patients from healthy controls. Our study contributes to this diverse landscape of research by introducing a neural network-based approach focused on uncertainty estimation, offering a different perspective on the classification challenges in Alzheimer's disease.

In this study, we introduce a novel approach that bridges the gap between traditional neural networks and Bayesian neural networks (BNNs) in the context of AD classification. Our method innovatively converts a standard neural network into a BNN post-training. This unique strategy significantly reduces the computational complexity and resource demands typically associated with BNNs. Moreover, our approach enables efficient uncertainty estimation in neural network predictions without the need for ensemble techniques. Our methodology aims to streamline the transition from research to clinical practice in Alzheimer's disease classification, eliminating the complexity typically associated with training for uncertainty estimation. Initially, we train a standard Convolutional Neural Network (CNN). During the inference phase, this CNN is transformed into a probabilistic network akin to a Bayesian network. This transformation is paired with a rejection approach based on user-defined thresholds. This system is designed to autonomously classify cases where the network is reasonably confident while directing all other cases for human evaluation, thus ensuring reliability and practicality in clinical applications. This advancement offers a more practical and resource-efficient solution for medical diagnostic applications, particularly in settings with limited data and computational resources.

## 2 Methods

In this section, we describe the dataset and briefly revisit the theory behind Bayesian neural networks that justify our approach.

### 2.1 Dataset

We selected a subset of 376 cases from the ADNI (Petersen et al., 2010) dataset, composed of cases labeled as both healthy and AD and employed only the Magnetization Prepared Rapid Gradient Echo (MPRAGE) T1-weighted image. T1-weighted (T1w) images were co-registered to the MNI template using linear initialization and a non-linear warp, after which the Jacobian Determinant (JD) maps were computed by isolating the non-linear part of the deformation field which takes the images from native space to standard space. Finally, we mask the deformation maps using the standard MNI brain mask. Registration procedures were performed using the ANTs package (Avants et al., 2008).

**Figure 2:**
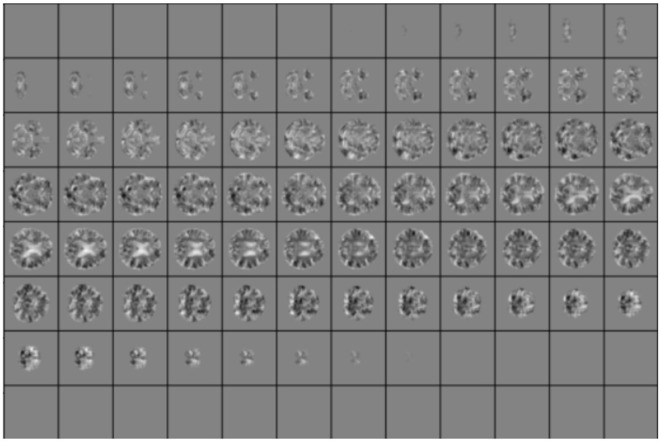
Example of slices for one random case in the test set. Local Jacobian Determinant images are normalized in the range [0, 1].

The high-dimensional non-linear transformation (symmetric diffeomorphic normalization transformation) model was initialized through a generic linear transformation consisting of center of mass alignment, rigid, similarity, and fully affine transformations followed by non-linear warps (metric: neighborhood cross-correlation, sampling: regular, gradient step size: 0.12, four multiresolution levels, smoothing sigmas: 3, 2, 1, and 0 voxels in the reference image space, shrink factors: 6, 4, 2, and 1 voxels. We also used image histogram matching before registration and data winsorization with quantiles 0.001 and 0.999. The convergence criterion was set to be as follows: the slope of the normalized energy profile over the last 10 iterations < 10^−^8). Co-registration of all scans required ~19,200 h of CPU time on a high-performance parallel computing cluster. Our final dataset consisted of 376 JD images, evenly distributed between AD and healthy cases. The data set was split into 80 (train)/20 (test) fashion, normalized globally, and cropped to a size (96, 96, 96). An example case is shown in Figure 2.

### 2.2 Bayesian neural networks and uncertainty estimation

We briefly recap the theory behind Bayesian neural networks and then describe the architecture of our model and the training procedure.

The idea is that instead of estimating w^*^, which minimizes the cost function, we learn a weight distribution. This is equivalent to an infinite ensemble approach, which allows us to estimate the variance of the prediction by sampling a slightly different neural network each time we perform inference. Instead of learning w^*^, we learn the posterior *p*(*w*|*D*), where *D* represents the incoming data. Here, our aim is to perform inference as the average of different neural networks, as described in Equation (1).


(1) $$p(\hat{y}|D)=\int_{w}p(y|w)p(w|D)dw=E_{p(w|D)}(p(y|w))$$

where *p*(ŷ|*D*) is the probability of obtaining the prediction *y* given the data *D*, *p*(*y*|*w*) is the conditioned probability of obtaining *y* given the weights of the network *w*, and *p*(*w*|*D*) is the posterior probability of having weight *w* given the data *D* as shown in Equation (2).

To perform this computation, we need the posterior *p*(*w*|*D*), which can be rewritten using the Bayes theorem. *p*(*w*|*D*) can be expressed by the likelihood *p*(*D*|*w*) and the prior *p*(*w*). However, the normalization term in the denominator is aslo needed albeit computationally intractable. There are several approaches to overcome this issue. One popular approach is variational inference, which tries to estimate *p*(*w*|*D*) by approximating this distribution with a parametrized distribution *q*_ϕ_(*z*) that minimizes the Kullback–Leibler (KL) divergence with the target distribution. Monte Carlo approaches are also available, with sampling points that match the required distribution as described in the studies mentioned in Kingma and Welling (2014), Blundell et al. (2015), Gal and Ghahramani (2016), and Lee et al. (2023).

The Monte Carlo approach is computationally extremely intensive, while using variational inference requires changes in the objective function that accomplish a new task described by a modification in the loss function. In this case, the standard loss function is augmented with the KL divergence between the distribution of the weights and the chosen prior, which can make training unstable and longer. Other methods aim to tackle the issue of estimating uncertainty. These include a variety of techniques, such as using ensembling approaches (Ganaie et al., 2022), dropout (Gal and Ghahramani, 2016), and others (Abdar et al., 2021), including limited-cost laplace estimation like in Daxberger et al. (2021) using efficient computations of Hessian. However, most of those methods require higher computational costs, are not scalable, and modify the objective function.

In this study, assuming that optimal values of w^*^ can serve as the center of Gaussian distribution; little deviation around these optimal values can represent similar networks, and this approach turns a standard neural network into a Bayesian neuroal network without the need for any added complexity at training time. Under the general assumption that the test distribution is comparable to the training distribution, small changes in the weights of the last layer should not result in divergent outcomes since optimal weights are expected to be in a global or local minimum. In addition, in real-world applications, it is common to have a slight distribution shift between training and test data, and thus, the weights often cannot represent a minimum for all or part of the test set. Our modifications enable the network to estimate the uncertainty and hence the shift from the minimum. If the uncertainty between slightly different network predictions is high, the network's predictions are not stable, and that specific case can be flagged for expert human evaluation.


(2) $$p(w|D)=\frac{p(D|w)p(w)}{\int_{w^{\prime }}p(D|w^{\prime })p(w^{\prime })dw^{\prime }}$$

### 2.3 Neural network architecture

**Figure 3:**
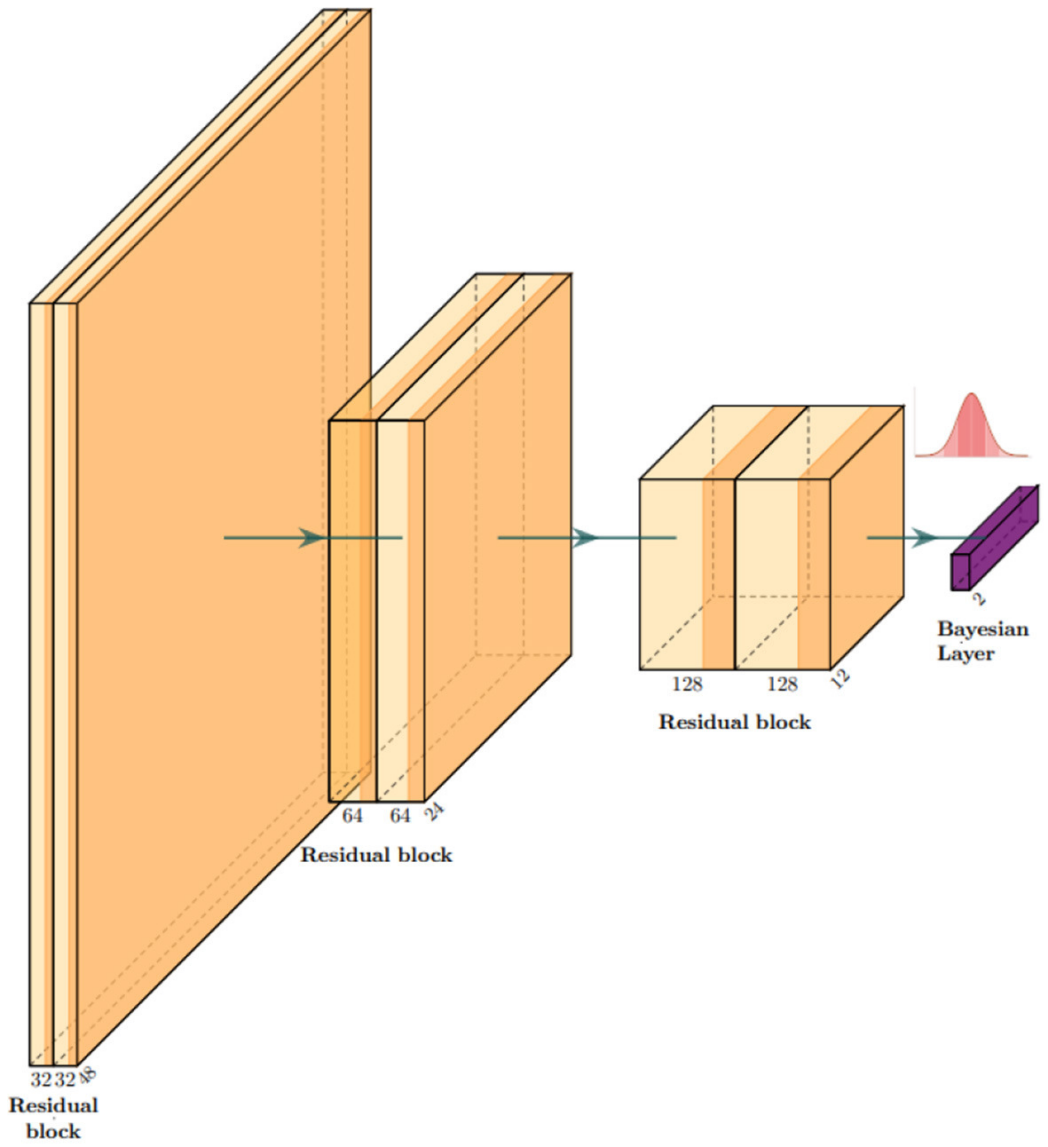
Architecture overview: our model is a classifier based on residual convolutional blocks. Each block is composed of two depth-wise separable convolutional layers to keep the number of parameters as low as possible to process 3D images. In correspondence with the gaussian distribution symbol, we sample the network classifier weights $w\sim N\left(w^{*},s\right)$.

Our base model is a residual convolutional neural network based on depth-wise separable convolutions, which we implemented to reduce the number of parameters and the risk of overfitting. 3D depth-wise separable convolutions are based on an *ad hoc* PyTorch implementation, using grouped convolutions with the group number set to the same value as the number of input channels, followed by a point-wise convolution with output channels. In other words, convolutions are first learned channel-wise, and then, information about the interaction between channels is taken into account by the second depth-wise convolution for each point. This reduces the number of parameters from *COK*^3^ to *C*(*K*^3^+*O*). Here, *C* is the number of input channels, *K* is the 3D kernel size, and *O* is the number of output channels. Our model consists of three residual blocks, and each block is composed of two depth-wise separable convolutions with a PReLU activation function (He et al., 2015). Each block halves the side dimension of the images. A flattening layer is followed by a linear layer for the first part of the training and then turned into a Bayesian linear layer replacing optimal values with Gaussian distributions. We used the Adam optimizer (Kingma and Ba, 2014) with a learning rate of 3*e*−4 and trained our model for five epochs. All implementations were built in Python, using Pytorch, Monai (Cardoso et al., 2022), and Torchbnn libraries (Lee et al., 2023). Figure 3 shows a scheme of the whole architecture.

After training, the weights of the last layer are replaced by a set of Gaussians w* → *N*(w*, *s*), and we set *s* = 0.01 in our experiments. This is an empirical choice that can be tuned in for specific purposes, always ensuring that the resulting weight perturbations remain small. At each forward pass, the network processes information in the standard way until it reaches the last layer. Here, a set of weights is sampled from $w\sim N\left(w^{*},s\right)$.

### 2.4 Experiment

Our model is trained to classify AD and healthy cases from JD images. Inference is run on the test set with *N* = 100, sampling the weights from their distributions as described above, after which the *softmax* of the output is computed to obtain the probabilities and aggregate the results using means and standard deviations. With this information, we explored a set of thresholds on standard deviation to examine the resulting variation of performances. Each estimation with a standard deviation with over the predetermined threshold is rejected and excluded from the performance estimation. The threshold can be set arbitrarily according to used needs. If higher accuracy is required, one can set a small value for the threshold *t*. In this case, we will have fewer data accepted for estimation and more “rejected” cases to be reviewed manually. On the other hand, most or all of the test sets can be retained if one is able to accept more misclassified cases.

The Algorithm 1 and 2 describes the entire procedure.

**Algorithm 1:**
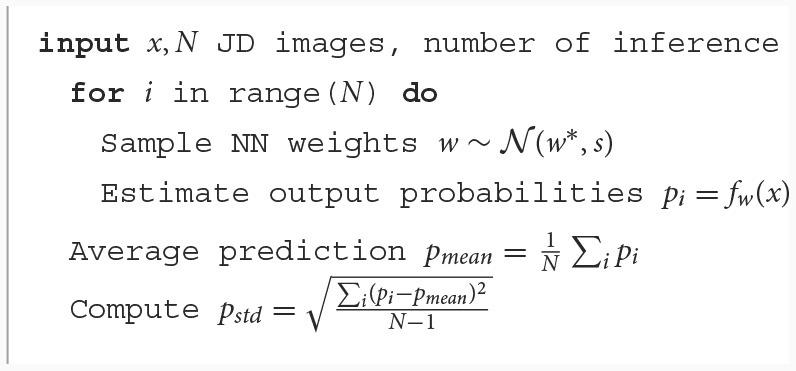
Inference.

**Algorithm 2:**
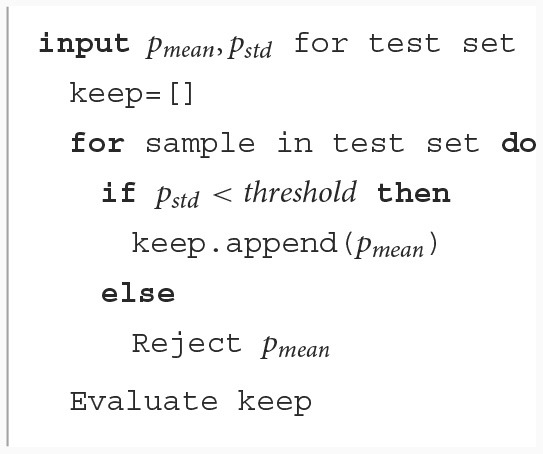
Reject procedure.

### 2.5 Explainability

To visualize the portion of the images that was weighted mostly by our model, we used the trulens library (Leino et al., 2018) implementation of integrated gradients, as shown in the study mentioned in Sundararajan et al. (2017). A baseline *x*_0_ image—usually a tensor of zeros—is generated, and a set of interpolated images are computed according to the formula *x*_*i*_ = *x*_0_+α(*x*−*x*_0_) where *x* is the actual image that we are trying to explain and α is a linearly spaced set of coefficients in [0, 1]. All those images are passed to the network, and the gradients along the path to the chosen class are collected and integrated. The images are smoothed with a 3D Gaussian kernel with σ = 4 to reduce noise in the procedure and keep the values above the 95 percentile to obtain a mask for the most important regions. The procedure is repeated 10 times, after which the attribution masks are averaged.

## 3 Results

As a baseline, we tested the standard neural network (i.e., without last layer substitution). In this case, in 100% of the test set, we obtained an accuracy of 0.86, an F1 score of 0.87, precision of 0.86, and recall of 0.86 with an AUC of 0.938.

**Figure 4:**
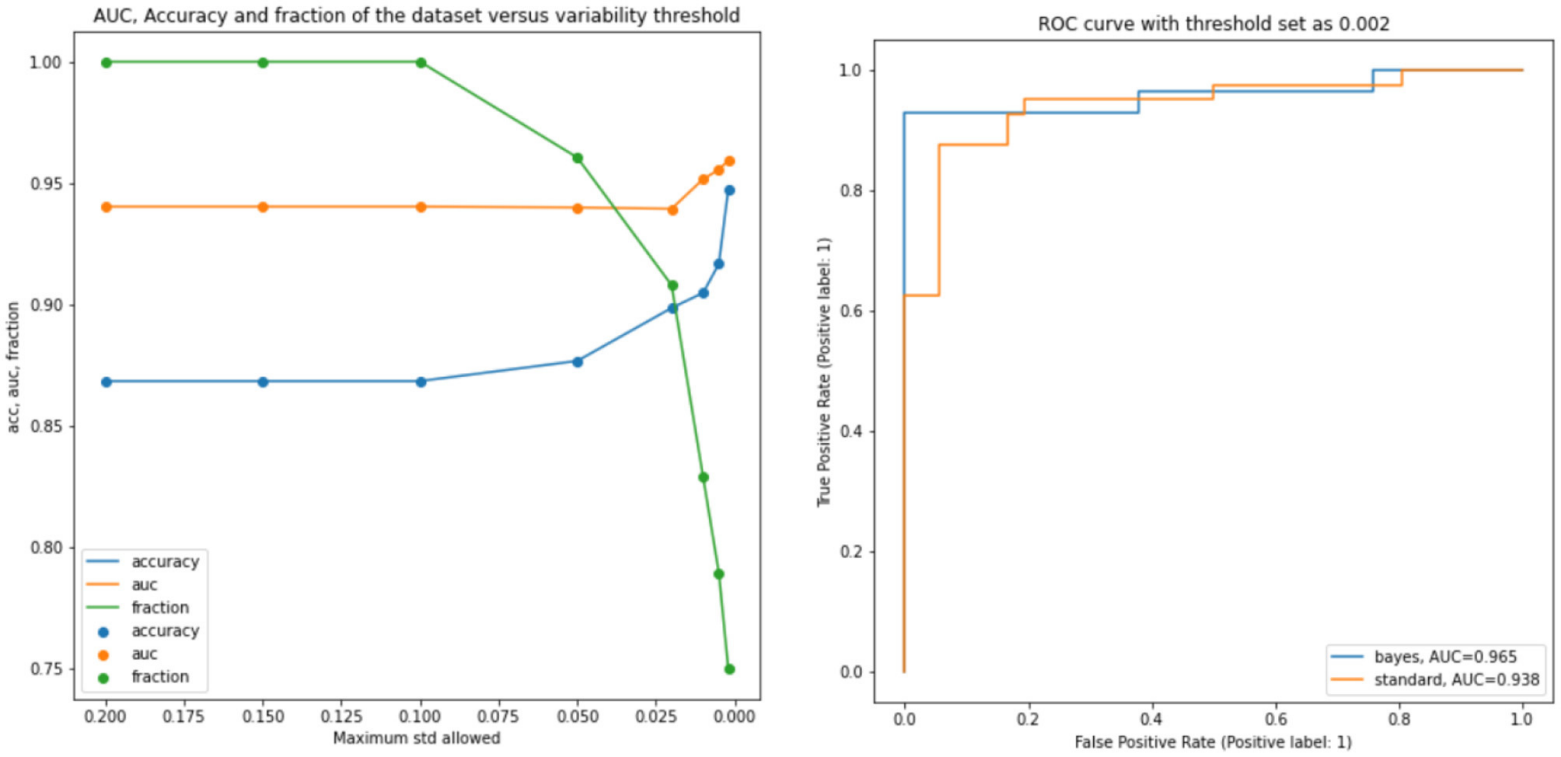
Results: the **left figure** accuracy (blue), AUC (orange), and the fraction of the test dataset (green) are shown as functions of the threshold. **Right figure**: AUC for the standard model and a selected bayesian model with a threshold of 0.002.

**Figure 5:**
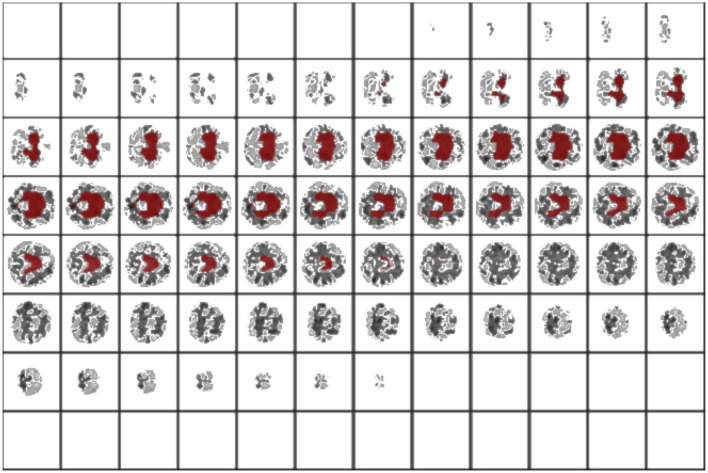
Results: integrated gradients. This is an interoperability method to look at the most influent areas for prediction. In this case, the model focuses on the ventriculi of the brain, an area which is involved in neurodegeneration.

Successively, our approach was tested as a function of *t* (i.e., the maximum standard deviation accepted for the class with the highest probability, see above). We computed the area under the receiver operating characteristic curve and the fraction of the retained test dataset for each threshold. In Figure 4 and Table 1, the results are reported as a function of the threshold value. We can clearly see two opposite trends, where reducing the threshold result in increases in accuracy and AUC, while the employed fraction of the test set naturally decreases, since the model is rejecting the predictions whose associated uncertainty exceeds the threshold. In our use case, we observed the best results with a threshold of 0.002, which retains 75% of the dataset and reaches an accuracy of 0.95 and AUC of 0.96. Figure 5 shows the final explainability masks generated by averaging integrated gradients for randomly chosen AD and healthy cases in the test set. It appears that the model focuses on different areas of the lower brain and, in particular, the ventricular spaces, whose deformation/enlargement due to atrophy is known to be associated with AD (Nestor et al., 2008; Ott et al., 2010).

**Table 1:** Results: accuracy, AUC, and the fraction of the test dataset with uncertainty behind the threshold.

**Threshold**	**Accuracy**	**AUC**	**fraction**
0.002	**0.947**	**0.959**	0.750
0.005	0.916	0.955	0.789
0.01	0.904	0.951	0.829
0.02	0.898	0.939	0.907
0.05	0.876	0.939	0.960
0.10	0.868	0.940	1.0
0.15	0.868	0.940	1.0
0.2	0.868	0.940	1.0

Bold values indicate the highest (best) values.

## 4 Discussion

In this study, we introduce a simple and yet effective way to modify a standard neural network into one that is able to accompany predictions with uncertainty estimates. In a wide range of application fields, for example, the medical industry, the readiness to accept forecasts by autonomous devices, especially those based on black-box algorithms such as neural networks, is crucial. The contribution of such tools to medical diagnosis, screening, and triaging is constrained by ethics and responsibility. We argue that tools that can have different behavior controlled by the user needs (e.g., setting higher or lower thresholds on uncertainty of predictions) constitute an important step in this direction.

Here, we introduce a novel application of this concept for Alzheimer's disease classification using neural networks, which is tailored to align with practical needs in a hospital setting. Our method is designed as a potential screening tool, offering high efficiency without additional computational burden during training. Importantly, it can be easily modulated to suit specific hospital requirements. During the inference phase, our network employs an uncertainty estimation mechanism. This allows for the identification of cases where the model's confidence in its prediction is below a pre-defined threshold. In such instances, rather than making a potentially unreliable decision, the network “asks for human evaluation.” This means that these complex cases are flagged for review by medical professionals, ensuring a higher reliability in diagnoses. In our experimental setup, as presented in Figure 1, we can automatically indentify a fraction of the test dataset that consists of samples about which the network was unsure. These were intentionally set aside, and the network proceeded to automatically evaluate the remaining less ambiguous samples. This approach simulates a real-world scenario, where challenging cases would be highlighted and forwarded to medical staff for further examination. It underscores our commitment to accuracy and safety in medical diagnosis, acknowledging the critical role of human expertise in conjunction with advanced AI technology.

Furthermore, in our methodology, as in any other deep learning medical imaging studies, the curation of the dataset and preprocessing is a crucial step for the success of automatic screening methods. Following previous research, we partially preprocessed the information for the network computing the Jacobian Determinant images to highlight structural differences between healthy and pathological cases. Although we employed the state-of-the art Advanced Normalization Tools (ANTs) for registration, there remains an inherent limitation in this process, since the registration could be imperfect and sometimes generate artifacts. While we meticulously vetted and excluded visually suboptimal cases, the registration step could still introduce noise and outliers. This potential source of variance is an important consideration in interpreting our findings. Our study highlights the need for continuous improvement in registration techniques and underscores the significance of considering these factors in similar neuroimaging-based AI studies.

Features such as uncertainty estimation and automatic rejections can improve the translation of research to clinical predictive models. Rather than completely replacing humans in evaluation, AI can support extremely useful recommendation systems and powerful tools to reduce workload in an efficient way for, example, medical professionals. We proposed a method that turns a classical neural network into a Bayesian neural network, hence endowing the model with the ability to estimate the uncertainty associated with predictions. Unlike other methods, we do not use optimized Gaussian distributions, but rather empirically narrow Gaussian distributions centered on the best weight value determined by the conventional training procedure. Our objective is to enable the network to explore the loss landscape on the test distribution data in order to identify situations where the predictions are not stable with minor weight perturbations, indicating that the loss is not minimized due to distribution shift, and hence, predictions cannot be trusted. This is based on the intuition that if the model's weight configuration minimizes the loss on the training set, predictions on unseen images should remain consistent even after small changes in the network's weights, as long as there is no change in the data distribution. In turn, this is motivated by the assumption that loss function's local landscape is relatively flat around the minimum. However, in the presence of slight differences between the training and test data distributions, unreliable predictions can emerge. Our approach addresses this by using a distribution of slightly different neural networks and their standard deviation across predictions as an approximation of the local Hessian, hence providing an estimation of uncertainty at inference time without adding significant computational complexity. We also incorporated a rejection method based on thresholding the estimated uncertainty, which has resulted in a global performance increase (which amounts to reducing probably misclassified cases as they are associated with higher uncertainty) over a considerable portion of the test dataset. Additionally, by exclusion, this system can select cases to be recommended for expert human evaluation when the uncertainty is above the threshold.

## 5 Conclusion

We built a Bayesian-based neural network method capable of estimating variability in predictions by simulating sampling from an infinite neural network ensemble. We used the estimated variability combined with a rejection method to retain only the fraction of the dataset that the model can classify with an uncertainty below the threshold and showed that this procedure can improve the accuracy from 0.86 to 0.95 (while retaining 75% of the test) when discriminating for AD from healthy cases based only on brain morphometry. Using integrated gradients, we also found that our model focuses on areas of the brain that are consistent with the clinical presentation of AD, in addition to highlighting previously unexplored areas in the lower part of the brain.

## Data availability statement

Publicly available datasets were analyzed in this study. This data can be found here: https://adni.loni.usc.edu/.

## Ethics statement

The studies were conducted in accordance with the local legislation and institutional requirements. Written informed consent for participation was not required from the participants or the participants' legal guardians/next of kin in accordance with the national legislation and institutional requirements.

## Author contributions

MF: Conceptualization, Formal analysis, Investigation, Methodology, Software, Visualization, Writing – original draft, Writing – review & editing. TB: Investigation, Writing – review & editing. NT: Funding acquisition, Supervision, Writing – review & editing.
