# Supplementary material for: Enabling uncertainty estimation in neural networks through weight perturbation for improved Alzheimer's disease classification
Source: Front Neuroinform. 2024 Feb 6;18:1346723. doi: 10.3389/fninf.2024.1346723 (PMC10876844; doi:10.3389/fninf.2024.1346723)
Supplement: Supplementary file 1 [file Presentation_1.pdf]

Appendix

Extended Experiments on MedMNIST Dataset

We extended our methodology to the MedMNIST dataset, a comprehensive benchmark for medical image classification tasks. This dataset includes various 3D medical images, allowing us to assess our method's performance in diverse medical screening contexts. The MedMNIST dataset is a diverse, standardized resource for biomedical image analysis, comprising both 2D and 3D datasets. It includes a wide range of medical imaging modalities, formatted into small, uniform sizes for ease of use in machine learning applications. This dataset collection facilitates research across various medical imaging tasks, providing a valuable benchmark for evaluating the generalizability of machine learning algorithms in the medical domain. We compared the classical approach with our conversion to bayesian and rejection-based approach, applying a threshold that discards at least 10% of the data. This threshold serves as a common criterion across datasets and simulates the decision-making process in a hospital setting, where thresholds can be adjusted based on specific requirements. The results, summarized in the table below, demonstrate significant improvements in accuracy and F1 score across different medical datasets. The table presents results for various datasets within MedMNIST. 'Base Acc' and 'Base F1' represent accuracy and F1 scores using the classical approach. 'Final Acc' and 'Final F1' are the scores after applying our rejection-based method. 'Improvement' columns show the relative enhancement in performance, and 'Fraction Predicted' indicates the percentage of data predicted based on uncertainty, while for the others the network in not sure enough, so doesn't predict and in a real scenario will ask for human evaluation. Our findings indicate significant improvements in accuracy and F1 scores across most datasets, with accuracy improvements up to 20%.

Dataset	Base Acc	Base F1	Final Acc	Final F1	% Imp. Acc	% Imp. F1	Fraction Predicted
OrganMNIST3D	0.792	0.824	1.000	1.000	26.29%	21.35%	54,92%
NoduleMNIST3D	0.861	0.799	0.952	0.851	10.58%	6.52%	60.97%
VesselMNIST3D	0.908	0.657	0.953	0.488	4.96%	-25.75%	66.75%
AdrenalMNIST3D	0.755	0.443	0.940	0.485	24.50%	9.31%	33.56%
SynapseMNIST3D	0.719	0.493	0.790	0.460	9.96%	-6.68%	70.45%
FractureMNIST3D	0.425	0.314	0.500	0.333	17.65%	6.02%	0.83%

Table 2: Extended Experiment Results on MedMNIST Dataset. 'Base Acc' and 'Base F1' represent accuracy and F1 scores using the classical approach. 'Final Acc' and 'Final F1' are the scores after applying our rejection-based method.

Notably, this positive enhancement was achieved while retaining predictions for more than half of each dataset. This demonstrates the efficacy of our method in increasing prediction reliability without significantly reducing the data used for decision-making in various medical screening tasks. These results highlight the adaptability and robustness of our method across various medical screening tasks. The improvements in accuracy and F1 scores across most datasets confirm the potential of our approach in diverse medical contexts. We note that the method's effectiveness varies with different datasets, reflecting the unique

challenges each medical screening task presents.

Comparison between depth-wise convolutions and classical 3D convolutions

We conducted an ablation study replacing depth-wise separable convolutions with standard 3D convolutions in our architecture. This experiment aimed to evaluate the impact of convolution type on performance, particularly in a small data regime typical of clinical environments. The model with normal convolutions comprised approximately 5 million parameters, a significant increase from the 900k parameters in our original model with depth-wise separable convolutions. Despite the larger model size, our findings revealed a decrease in performance. The table from the original experiment shows results with depth-wise separable convolutions, with accuracies as high as 0.947 and AUC scores up to 0.959. In contrast, the experiment with normal convolutions showed a noticeable decline in both accuracy and AUC scores across various thresholds, with the highest accuracy recorded at 0.85 and a corresponding AUC score of 0.878. This comparative analysis underscores the efficacy of depth-wise separable convolutions in our context, balancing model complexity with performance, especially in scenarios with limited data availability.

**Threshold (%)**	**Normal Convolution**			**Depth-wise Convolution**		
	**Accuracy**	**AUC**	**Fraction (%)**	**Accuracy**	**AUC**	**Fraction (%)**
0.002	0.85	0.878	77.63	**0.947**	**0.959**	75.0
0.005	0.84	0.869	80.26	**0.916**	**0.955**	78.9
0.01	0.83	0.862	84.21	**0.904**	**0.951**	82.9
0.02	0.82	0.860	86.84	**0.898**	**0.939**	90.7
0.05	0.81	0.863	90.79	**0.876**	**0.939**	96.0
0.10	0.81	0.857	96.05	**0.868**	**0.940**	100.0
0.15	0.81	0.851	98.68	**0.868**	**0.940**	100.0
0.20	0.82	0.848	100.0	**0.868**	**0.940**	100.0

Table 3: Comparison of Normal Convolution and Depth-wise Convolution Approaches
